# Agroecology: protecting, restoring, and promoting biodiversity

**DOI:** 10.1186/s12862-023-02140-y

**Published:** 2023-07-04

**Authors:** Jessica Knapp, Andrea Sciarretta

**Affiliations:** 1 Department of Botany, School of Natural Sciences, Trinity College Dublin, Dublin, Ireland; 2 Department of Agricultural, Environmental and Food Sciences, University of Molise, Campobasso, Italy

## Abstract

The global food system is the predominant driver of biodiversity loss. Consequently, there is an increasing need to transition towards more sustainable and resilient agri-food systems to protect, restore and promote biodiversity. To help address this issue, BMC Ecology and Evolution has launched a new article Collection on agroecology.

## Background

Globally, agricultural land continues to expand and intensify to meet rising food, fibre and energy demands [1, 2]. Although successful in increasing yields, the loss and simplification of natural habitats combined with the replacement of many biological functions with artificial inputs has negatively affected the resilience and productivity of agricultural systems [3]. These anthropogenic drivers, and the implications for food security, make biodiversity decline and conservation a significant issue in agricultural landscapes.

Sustainable farming requires synthetic inputs to be complemented or replaced with ‘environmentally friendly’ practices that manage regulating and supporting ecosystem services to enhance agricultural productivity [2]. Doing so often requires a transition from reactive to preventive measures such as diversifying crop production, shown to enhance multiple ecosystem services, such as pollination and nutrient cycling, without compromising yield [4]. Advances in crop protection have also allowed for the development of numerous alternative methods to chemical pesticides, chemical fertilisers, and antibiotics in agroecosystems, guided mainly by integrated pest management principles. Over the years, they have brought important new applications and success stories, such as the widespread use of semiochemicals for the monitoring and control of many insect pests [5], the use of microorganisms as biocontrol agents [6] or the augmentation of biological control by the action of natural enemies [7]. These methods, in combination with landscape and field management and the application of sampling, monitoring and thresholds, can improve yields [8] and minimise negative impacts from intensive agriculture, such as habitat loss, reduced soil fertility, and pesticide poisoning of non-target species [2, 9].

However, these management practices may be challenging to implement because they require in-depth knowledge of the functioning of trophic networks, in combination with human-mediated stressors, over relevant spatial and temporal scales [10, 11]. Robust indicators to quantify management impacts are needed [10, 11], especially since effects are often variable, depending on species ecologies and, for highly mobile organisms like pollinators, the complexity of the habitat surrounding a crop field [12]. Understanding and generalising this context-dependency can improve decision-making by determining a management practice’s optimal location in the landscape and can help determine practices that benefit a particular species’ conservation or ecosystem service. This knowledge is essential for precipitating a more nuanced understanding of agroecology. How landscape-level processes moderate biodiversity patterns and ecosystem functioning and how they change and interact at different scales are still underexplored [13].

Developing a deeper understanding of species ecology and their ecosystem functions, especially over larger spatiotemporal scales [3] and with under-researched agricultural systems, geographic areas, taxa, and ecosystem functions such as belowground inhabitants and their effects on soil fertility [10, 14] is critical for determining a strong evidence base for the benefits of sustainable farming. Furthermore, identifying synergies between ecosystem services such as pest control and pollination and agronomic and economic benefits may increase farmer interest, offering the potential to upscale agroecological practices, especially if they align with existing agricultural practices [15].

There is an increasing need to transition towards more sustainable and resilient agri-food systems to protect, restore and promote biodiversity. Ambitious sustainability targets such as those agreed upon via the UN (United Nations) Biodiversity Conference (COP 15) and the European Farm to Fork strategy (The European Commission, 2019) offer vast potential to benefit biodiversity and ecosystem services in agricultural landscapes. However, meeting these targets depends on functional and sustainable management practices underpinned by scientific evidence that is scalable to different environmental contexts.

In support of the UN’s Sustainable Development Goals, BMC Ecology and Evolution welcomes submissions to a new article Collection on agroecology. The Collection aims to bring together research from around the globe on the development and implementation of sustainable agricultural practices that promote healthy ecosystems, upscaling agroecological practices, the impact of agroecology practices on beneficial species, the role of consumers in agro-ecological food systems, matter and energy flows in agroecosystems, agroecology practices and soil management, the ecological management of plagues and diseases, ecological restoration in agricultural landscapes, the possible use of agroecology to achieve carbon neutrality and agroforestry.

## Data Availability

Not applicable.

## Abbreviations

COP15: Conference of the Parties to the UN Convention on Biological Diversity
UN: United Nations
